# Usefulness of Telemonitoring in Respiratory Deterioration in a Patient Treated With Home High-Flow Nasal Therapy

**DOI:** 10.1155/crpu/9535168

**Published:** 2025-10-09

**Authors:** Jean-Michel Arnal, Thomas Obert, Aude Garnero

**Affiliations:** ^1^Intensive Care Unit and Home Ventilation Unit, Centre Hospitalier Intercommunal de Toulon La Seyne sur Mer, Toulon, France; ^2^SOS OXYGENE VAR, Cuers, France

**Keywords:** chronic obstructive pulmonary disease, high-flow nasal cannula oxygen therapy, obstructive sleep apnea, overlap syndrome, patient engagement, telemonitoring

## Abstract

A 63-year-old male with overlap syndrome had high-flow nasal cannula (HFNC) therapy added to his treatment in May 2023 after reporting reduced exercise tolerance due to dyspnea and recurrence of chronic bronchial congestion on existing therapy (noninvasive ventilation and appropriate drug treatment). HFNC therapy (Lumis HFT; ResMed) was started with prescribed usage of 2 h/day, a flow of 20 L/min, and a temperature of 37°C. Within 15 days, the patient noted improved clearance with thinner secretions, and then secretion volume decreased after 2 months. Six months after having HFNC connected to a telemonitoring platform (AirView), the homecare technician observed a sudden increase in HFNC usage (to > 4 h/day). After being contacted by the technician, the patient reported increased sputum production, low-grade fever, and exertional dyspnea. Evaluations found *Haemophilus influenzae*, and antibiotic therapy was started. The patient quickly improved and HFNC usage returned to the usual routine. Subsequently, the patient learned to adjust his HFNC usage based on the degree of bronchial congestion. This case report highlights the innovative value of telemonitoring as an early warning tool for exacerbations in patients receiving home high-flow nasal therapy, an aspect still scarcely documented in existing literature.

## 1. Introduction

High-flow nasal cannula (HFNC) therapy is recommended as a first-line treatment for acute hypoxemic respiratory failure in hospital settings and after extubation in intensive care units [1]. The use of HFNC at home has only taken place more recently. Daily humidification of inspired gases improves mucociliary clearance in patients with obstructive lung disease [2], with clinical benefits including reduced exacerbation frequency and lower hospitalization rates [3–6]. The Lumis HFT (ResMed) HFNC device is designed specifically for home use, providing lower flow rates than hospital devices; is easy to use for patients; and has remote monitoring capabilities. We present a case report where telemonitoring of home HFNC therapy allowed healthcare providers to detect a developing exacerbation early.

## 2. Case Presentation

The patient is a 63-year-old male with overlap syndrome, having both obesity-related obstructive sleep apnea (body mass index 31 kg/m^2^) and postsmoking chronic obstructive pulmonary disease (COPD; current nonsmoker, 50 pack-year smoking history). The patient's forced expiratory volume in 1 s (FEV_1_) was 26% of predicted. Chronic respiratory failure was diagnosed in 2020 during an acute exacerbation of COPD that required admission to the intensive care unit. At that time, home oxygen therapy (1.5 L/min) and noninvasive ventilation (NIV) were initiated (EPAP = 8 cm H_2_O; IPAP = 19 cm H_2_O; back‐up rate = 10 bpm), alongside treatment with a long-acting *β*_2_-agonist and a long-acting muscarinic antagonist. The patient was discharged home, where he lives alone, after completing a pulmonary rehabilitation program. He resumed daily walking for 90 min with ambulatory oxygen support. Nocturnal NIV usage was effective in maintaining normocapnia.

In May 2023, the patient reported reduced exercise tolerance due to dyspnea and recurrence of chronic bronchial congestion, with thick, hard-to-expectorate secretions. He was using NIV for 8 h and 15 min per night with normal arterial blood gases (PaO_2_ = 60 mm Hg; PaCO_2_ = 40 mm Hg). HFNC therapy (Lumis HFT; ResMed) was started with prescribed usage of 2 h/day, flow of 20 L/min, temperature of 37°C, and a size large nasal cannula [3]. Within 15 days, the patient noted improved clearance with initially more abundant and thinner secretions, and this was followed by a decrease in secretion volume after 2 months. The patient opted to continue HFNC therapy and self-adjusted the usage time to 1.5 h/day.

### 2.1. Intervention and Outcome

**Figure 1:**
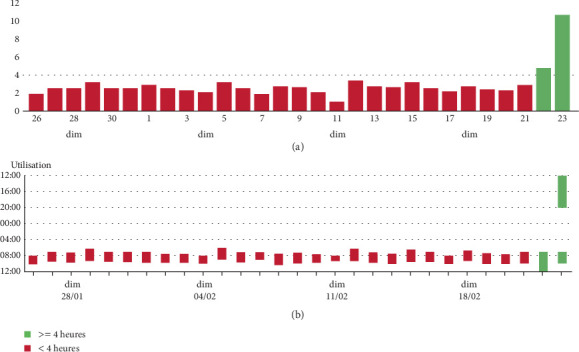
(a) Cumulative daily usage (hours per day). (b) Specific times of day the device was used. The green bars indicate when the daily use of HFNT suddenly increased.

**Figure 2:**
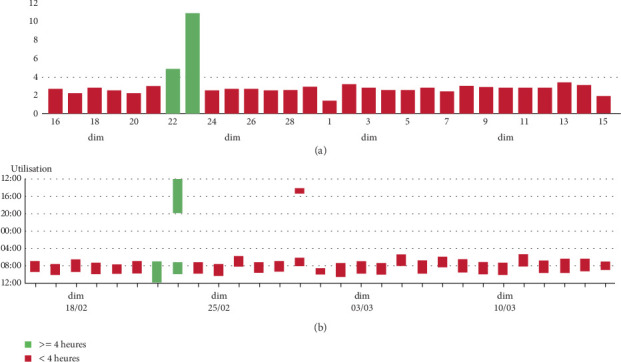
(a) Cumulative daily usage (hours per day). (b) Specific times of day the device was used. After 2 days with increased use, the patient resumed his usual daily use.

In August 2023, the HFNC device was connected to the AirView platform, enabling remote monitoring by the home care provider. In February 2024, the technician observed a sudden increase in HFNC usage to over 4 h/day on 2 consecutive days, with an unusually long afternoon session (Figure 1). Within the next 24 h, the technician contacted the patient, who reported increased sputum production, low-grade fever, and exertional dyspnea. The patient's physician was alerted and conducted a telephone-based Exascore [7] evaluation, suggestive of bronchitis without signs of clinical severity. Specifically, self-monitoring indicated that resting SpO_2_ remained above 92% while on his usual oxygen regimen [8]. Antibiotic therapy was initiated at home after testing showed the presence of *Haemophilus influenzae*. Given the acute increase in respiratory symptoms and the need for antibiotics, this episode meets criteria for a probable moderate COPD exacerbation according to GOLD, although pneumonia cannot be definitively excluded without chest imaging. Resting oxygen saturation remained at the patient's usual baseline, and no additional features suggested pneumonia, so imaging was not performed. The patient quickly improved and returned to his usual HFNC routine (Figure 2). After this episode, the patient learned to adjust his HFNC usage according to the degree of bronchial congestion.

## 3. Discussion

HFNC works by reducing anatomical dead space, reducing work of breathing and ventilatory burden. There is currently not a large volume of data on the use of HNFC in patients with overlap syndrome. One study suggested that it reduced nocturnal apneas and improved oxygenation [9], but no benefit in patients with overlap syndrome has been reported [10]. Our patient with overlap syndrome did benefit from HFNC therapy. However, the most interesting feature of this case was that HFNC telemonitoring data allowed the early detection of an acute moderate exacerbation of COPD, which led to timely intervention and possibly averted further clinical decline. Once a patient is familiar with the device, changes in HFNC usage duration could serve as an indicator of secretion burden, and sudden increases in usage may be an early sign of exacerbation.

Overall, telemonitoring adds value to home HFNC therapy by enabling early detection of clinical deterioration and guiding treatment adjustments based on real-life usage patterns. Empowering patients to self-modulate treatment contributes to individualized care and may prevent hospitalizations.

## Data Availability

The data that support the findings of this study are available from the corresponding author upon reasonable request.
